# PredatorNet: a clinically trustworthy deep learning framework for multi-class Alzheimer's disease classification using MRI

**DOI:** 10.3389/fnagi.2026.1835340

**Published:** 2026-07-06

**Authors:** T. Deenadayalan, S. P. Shantharajah

**Affiliations:** School of Computer Science Engineering and Information Systems, Vellore Institute of Technology, Vellore, India

**Keywords:** AD classification, calibration reliability, clinical trust score, deep learning technology, explainable AI, MRI, multi-class classification system, out-of-distribution detection system

## Abstract

MRI-based staging of Alzheimer's Disease (AD) requires not only high classification accuracy but also clinically reliable probability estimates, transparent evidence for decisions, and robustness to shifts in data distribution. We propose PredatorNet, a multi-class AD classification framework with three key modules: (i) EagleAttention, which uses spatial self-attention to focus on diagnostically salient regions; (ii) RegionImportance, which produces an explicit spatial weighting map to support interpretability; and (iii) WolfPackFusion, which fuses complementary feature pathways to improve prediction stability. The training process requires us to use weighted random sampling because we need to handle class imbalance. We establish the Clinical Trust Score (CTS) as a metric that evaluates clinical effectiveness through the combination of seven performance benchmarks. The test set results show PredatorNet achieves 96.64% accuracy with macro Receiver Operating Characteristic Area Under the Curve of 0.9950 and mean sensitivity of 0.9803 and mean specificity of 0.9876 across 1,280 MRI scans. The model shows strong calibration (Calibration Reliability = 0.9912) and stable explanations (Regional Importance Stability Index = 1.0000). The evaluation method achieves an Out-Of-Distribution (OOD) stability score of 0.2810, which demonstrates its ability to handle distributional shifts. The CTS results show a value of 0.9491 without anatomical weighting and a value of 0.8652 under stricter anatomy-based validation. The Gradient-Weighted Class Activation Mapping visualizations establish a connection between learned importance patterns and neuro-anatomical regions relevant to clinical practice, which supports clinically oriented reporting.

## 1 Introduction

AD progresses as a neuro-degenerative condition which results in cognitive impairment and deterioration of brain tissue (Jack et al., 2013; Wei et al., 2017; Ruan et al., 2016; Pathak and Kabra, 2024; Cummings, 2004). The early identification process enables medical personnel to begin treatment at the right times while monitoring the progression of the disease through specialized treatment approaches. The non-invasive method of MRI enables researchers to study brain structural changes which include hippocampal atrophy, cortical thinning, and ventricular enlargement (Sorour et al., 2024; Lehmann et al., 2011; Krajcovicova et al., 2019; Ledig et al., 2018; Alsubaie et al., 2024). Manual reading requires extensive effort because different observers interpret the results differently, creating a need for AI systems that provide scalable and consistent decision-making support systems (Jia and Lao, 2022; Khan et al., 2021; Klöppel et al., 2012; Kabir et al., 2021; Muhammed et al., 2023).

DL has enhanced AD detection using MRI imaging by improving methods for learning medical images, deploying attention systems, and integrating diverse data types. The study reports that specific methods achieve performance improvements through different approaches in ensemble transfer learning, which use Grad-CAM for explanation purposes (Mahmud et al., 2024) and through hybrid systems that combine CNN to extract features with traditional classifiers (AbdelAziz et al., 2024) and through post-hoc explanation methods, including LIME and Grad-CAM to show important areas of distinction (Junior et al., 2024). The combination of PET with MRI scanning techniques can lead to better sensitivity outcomes (Odusami et al., 2023; Mahmood et al., 2024), while attention-based fusion methods can improve the detection of important clinical signals (Golovanevsky et al., 2022). The combination of 3D CNNs with multi-scale attention systems has shown great potential to analyze three-dimensional patterns found in brain MRI according to studies done in Wu et al. (2022) and Liu et al. (2022).

The process of translation still faces multiple obstacles that need to be resolved. The studies provide partial clinical validation results together with incomplete tests of system strength which did not use established methods for performance assessment. The system needs to establish its operational capacity to handle multiple user groups and different operational environments which is particularly important during initial development stages when systems show only small changes (Feng et al., 2022). The reviews demonstrate that XAI methods (including LIME and SHAP) establish clinician trust while meeting regulatory requirements although researchers still lack methods to measure explanation accuracy through quantitative methods (Vimbi et al., 2024; Cremades et al., 2025). The use of segmentation-guided pipelines enables better anatomical examination but dataset bias problems together with insufficient external validation and fairness issues prevent their widespread adoption (Zubair et al., 2025; Javed et al., 2023).

The research presents **PredatorNet** as a solution to the identified gaps by introducing a *trust-aware deep learning framework* for multi-class Alzheimer's disease classification from MRI. Unlike conventional pipelines that optimize only predictive accuracy, PredatorNet is explicitly designed to jointly address **discrimination, calibration, interpretability, and robustness** within a unified architecture.

The name reflects its predator-inspired design: EagleAttention (a spatial self-attention refinement module) focuses on diagnostically salient anatomical regions, while WolfPackFusion (a multi-branch logit fusion mechanism) aggregates complementary decision pathways to improve stability and reduce variance. In addition, the RegionImportance module introduces a *learnable anatomical weighting mechanism* that enables explicit region-level attribution during training rather than relying solely on post-hoc explanation.

PredatorNet is built around three tightly coupled principles: (1) attention-driven representation learning for capturing fine-grained neuro-anatomical variation, (2) intrinsically integrated explainability through region-aware feature weighting, and (3) calibration- and shift-aware evaluation to support deployment-level reliability.

The main contributions are summarized as follows:

1. **Integrated trust-aware architecture:** We propose a modular framework combining spatial self-attention (EagleAttention), learnable region-based importance weighting (RegionImportance), and multi-branch fusion (WolfPackFusion). Unlike prior approaches where attention, explainability, and fusion are treated independently, PredatorNet integrates them into a single end-to-end trainable system.
2. **Intrinsic explainability with stability constraints:** Instead of relying only on post-hoc visualization (e.g., Grad-CAM), the model learns region importance maps during training, coupled with a smoothness regularization objective. This enables more stable and anatomically consistent explanations, quantified using the proposed Region Importance Stability Index (RISI).
3. **Multi-dimensional clinical trust evaluation:** We introduce a comprehensive evaluation protocol that jointly assesses discrimination (ROC-AUC), calibration reliability, explanation stability, and robustness to distributional shift (OOD), addressing a key gap in current medical AI validation practices.
4. **Composite Clinical Trust Score (CTS):** We propose CTS as a unified metric that aggregates multiple clinically relevant criteria into a single interpretable score. Unlike standard metrics, CTS captures the trade-off between predictive performance and trustworthiness, and is further extended with anatomy-aware validation (CTS-A).
5. **System-level contribution beyond accuracy optimization:** Rather than proposing a single novel module, PredatorNet contributes a *framework-level integration* that bridges model design and clinical evaluation, positioning it as a step toward deployable and trustworthy medical AI systems.

The paper is structured as follows. Section 2 examines prior work and highlights limitations in calibration, interpretability validation, and external robustness. Section 3 presents the PredatorNet architecture and the clinical trust assessment framework. Section 4 reports experimental and comparative evaluations, including ablation and trust-based analysis. Section 5 provides an in-depth interpretation of the results in a clinical context. Finally, Section 6 outlines limitations and future directions toward multi-center validation and deployment.

## 2 Related work

AD diagnosis research now uses AI/DL pipelines which learn data patterns from original data instead of using manually created features. Although the system demonstrates strong performance results, its application in everyday medical practice remains restricted because of insufficient external testing, which detects performance shifts, and the system's inability to provide measurable interpretation results. The document presents research studies between 2022 and 2025 which demonstrate trust-oriented assessment methods that this paper uses as its basis.

### 2.1 MRI-based deep learning for AD classification

The MRI serves as the main imaging method for Alzheimer's disease diagnosis because it shows structural patterns which include cortical thinning and hippocampal atrophy and ventricular enlargement that indicate different levels of disease progression (Ewers et al., 2011). The research findings demonstrate that the final outcomes result from three factors which include pre-processing choices and the selected population and the architectural design decisions made by researchers (Yu et al., 2023). The majority of CNN-based systems achieve high staging accuracy; however, they still depend on single-cohort evaluation which restricts their ability to reproduce results across different scanners and study locations (Sorour et al., 2024; El-Geneedy et al., 2023; Arafa et al., 2024). The need for methods that recognize site/scanner differences requires implementation during both model development and evaluation processes.

The process of increasing sample size in research studies leads to improved statistical power, yet it fails to eliminate both inter-site variability and equipment-dependent measurement errors (Mousavi et al., 2025; Wang et al., 2022). Recent studies have investigated hybrid CNN-transformer backbones that use MaxViT-style designs to model both short-term local texture patterns and extended spatial relationships (Chen et al., 2024; Aslan et al., 2025). The expansion of model capacity does not ensure accurate confidence measurements for clinical applications, which creates a need for evaluation methods that require calibration.

### 2.2 Hybrid and optimization-driven learning strategies

The standard approach involves combining deep feature extraction methods with traditional machine learning methods and direct optimization techniques to create better training results and improved decision-making accuracy (Xu et al., 2021). The study presents two examples which demonstrate CNN feature extraction processes that work together with KNN classifiers which researchers optimized through Bayesian optimization methods (Khan and Zubair, 2022; Lahmiri, 2023; Varfolomeev et al., 2019). Transfer learning continues as a popular method because it helps organizations cut down their data needs while making their systems run better and reach results faster (Mahmud et al., 2024; Saleem et al., 2022; Jassal et al., 2025; Di Nardo et al., 2021; Goldberger et al., 2000; Ksi ażek et al., 2024; Brya, 2020). The existing hybrid systems treat calibration together with interpretability as secondary features instead of treating them as essential design goals according to research findings (Albahri et al., 2023; Bhandari et al., 2020; Lungu et al., 2016; Chartrand et al., 2017; Alatrany et al., 2024; Zaitsev et al., 2015). The situation creates a demand for integrated models which handle trust-related requirements as primary design elements.

### 2.3 Explainable and interpretable AI in AD diagnosis

The clinical translation requires interpretable results because visual evidence needs to be established through saliency testing. The prediction explanation process uses model-agnostic tools to identify key areas which help to explain their results according to previous research (Alatrany et al., 2024; Fave et al., 2016). The field lacks standardized methods for validating explanation maps through comparison with anatomical references and neuropathological standards which creates doubt about their accuracy (Singh and Jain, 2023). The research develops learnable attribution mechanisms which undergo testing for both stability and anatomical alignment assessment.

### 2.4 Attention-based and cascade architectures

The goal of attention-based designs is to direct their representational strength toward essential diagnostic areas. Researchers discovered that cascade and hierarchical attention frameworks improve their capacity to identify initial structural changes (Khosravi et al., 2025; Lim et al., 2022). Most studies which examine attention mechanisms require testing on multiple datasets and need proper assessment of their calibration performance (Jermyn et al., 2015; Arif et al., 2023). Researchers need to evaluate attention mechanisms through assessment of calibrated confidence levels and explanation stability in addition to evaluation accuracy.

### 2.5 Multimodal and non-imaging approaches

The strength of multimodal approaches increases when they use multiple biomarkers together with their single MRI method (Qiu et al., 2022; Vrahatis et al., 2023; Deenadayalan and Shantharajah, 2024). Different research groups are studying non-imaging signals which include OCT, EHR-based risk prediction and survival-oriented modeling and behavioral biomarkers (Chua et al., 2025; Abuhantash et al., 2025; Deenadayalan and Shantharajah, 2025; Akter et al., 2025; Berenguer et al., 2018; Varghese et al., 2019; Larue et al., 2017; Fave et al., 2016; Mackin et al., 2015; Nardone et al., 2025). The efforts of all these initiatives lead to predictive systems that can work in a variety of fields. The key elements of scaling fusion techniques and building reliable reporting systems are still to be developed.

### 2.6 Early detection and systematic reviews

The identification process between CN individuals, MCI patients, and AD patients remains difficult because their biomarkers show similar patterns and their physical changes are hard to detect. The recent research introduces deep learning systems for early detection purposes (Mmadumbu et al., 2025), but the systematic reviews demonstrate three major problems which occur repeatedly: high dataset variability and insufficient external validation and restricted fairness evaluation (Zia-Ur-Rehman et al., 2025). The power of these findings drives us to conduct dependability tests which require results from various testing methods that supplement our accuracy assessments.

### 2.7 Comparative analysis

Table 1 overviews representative recent studies and synthesizes recurring limitations that motivate a trust-oriented evaluation framework.

**Table 1:** Comparative summary of AD classification studies (2022–2025).

References	Modality	Architecture	Explainability	Strength	Limitation
Aslan et al. (2025)	MRI	MaxViT Hybrid	No	Transformer integration	Interpretability gap
Hazarika et al. (2022)	MRI	CNN benchmarking	No	Comparative rigor	Single-dataset bias
Lahmiri (2023)	MRI	CNN + KNN + BO	No	Optimization-driven	Complexity overhead
Alatrany et al. (2024)	MRI	ML + XAI	Yes	Interpretability focus	Limited quantitative validation
Qiu et al. (2022)	Multimodal	Deep fusion	No	Heterogeneous integration	Fusion scalability
Khosravi et al. (2025)	MRI	Cascade Attention	Partial	Early-stage sensitivity	No cross-site validation
Mousavi et al. (2025)	MRI	DL (large-scale)	No	Large dataset	Domain variability
Abuhantash et al. (2025)	Clinical	ML Survival	No	Longitudinal modeling	Not imaging-based

### 2.8 Identified research gaps

The current methodology is not enough to fill the gaps between techniques that still exist:

1. **Clinical trustworthiness deficit:** The primary sources of uncertainty in machine learning emerge from three factors, which include domain shift, representation bias, and fairness requirements for gender role alignment.
2. **Interpretability validation gap:** Heat map explanations are presented without recourse to prior authentication or comparison with anatomical or neuropathological priors.
3. **Limited external validation:** Many studies rely solely on single-cohort evaluations, for example, the ADNI, which reduces the translational trust.
4. **Insufficient longitudinal modeling:** DL leveraging imaging data is seldom venture into time-lapse or personified malignancy.
5. **Fragmented multimodal fusion:** Although it is worth mentioning that centralized, widely agreed-upon multiple multimedia fusion frameworks are now emerging.
6. **Early-stage sensitivity constraints:** Structural defects in the molecule, though, might remain elusive, and their discriminatory scrutiny might pose exceedingly tough challenges.
7. **Deployment and regulatory considerations:** The research studies that exist for this topic only assess practical constraints, which include computation, clinician-in-the-loop validation, and compliance readiness.

### 2.9 Synthesis

The literature shows that both size of the dataset and the complexity of the model expand at a rapid pace while evaluation methods for research work remain unstandardized. The framework needs to assess all components that include discrimination, calibration, robustness to distribution shift, and validated explainability. AI-assisted AD diagnosis research requires this gap closure to transform from prototype development into operational systems. PredatorNet and its trust assessment protocol that meets operational needs are introduced in the upcoming section to fulfill these requirements.

## 3 Proposed methodology: PredatorNet for trustworthy AD classification

### 3.1 Overview of the proposed framework

The proposed methodology introduces **PredatorNet**, a deep neural architecture developed for clinical applications that performs AD classification from structural MRI scans. PredatorNet develops clinical trustworthiness through its interpretability elements, calibration reporting system and its robustness testing procedure while traditional CNN systems focus only on achieving accurate results.

The framework consists of four modules which work together as one system.

1. Data pre-processing and augmentation.
2. Trust-aware architecture design with multi-level attention.
3. Imbalance-robust training strategy.
4. Comprehensive trustworthiness evaluation.

The modules work to solve major problems that occur in medical imaging decision support systems, including high intra-class variability, severe class imbalance, limited model interpretability, and overconfidence under distribution shift.

**Figure 1:**
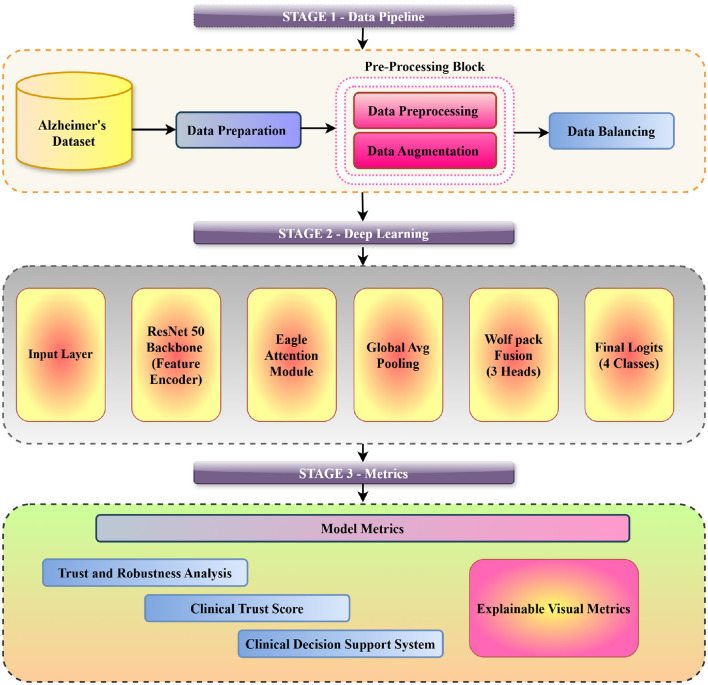
PredatorNet architecture overview. The process includes multi-scale convolutional feature extraction, hybrid spatial channel attention, trust calibration layers, and uncertainty-aware classification, which ensures a clinically valid diagnosis for AD.

The end-to-end pipeline is illustrated in Figure 1.

### 3.2 Dataset description

The dataset used in this study is an Alzheimer's Disease MRI dataset designed to support deep learning-based diagnosis of cognitive impairment stages. The dataset consists of structural brain MRI scans stored in JPG format with a standardized resolution of 256 × 256 pixels. The data is derived from a combination of publicly available sources (e.g., ADNI) and curated clinical datasets, enabling diverse representation across disease stages.

Each image is associated with metadata including participant-level information such as age, gender, and diagnosis history, enabling potential future extensions toward multimodal learning and clinical analysis.

#### 3.2.1 AD stage classification

The dataset is annotated into four clinically relevant stages of Alzheimer's Disease progression (Table 2).

**Table 2:** AD stage labels used for classification.

Label	Abbrev.	Description
NonDemented	ND	No observable cognitive impairment
VeryMildDemented	VMD	Early-stage cognitive decline
MildDemented	MD	Noticeable cognitive impairment
ModerateDemented	MOD	Advanced stage of dementia

#### 3.2.2 Dataset composition and split

The dataset is provided with predefined training and test subsets. Table 3 summarizes the class distribution.

**Table 3:** Dataset composition (number of images) by split and class.

Class	Train	Test
NonDemented (ND)	2,560	640
VeryMildDemented (VMD)	1,792	448
MildDemented (MD)	717	179
ModerateDemented (MOD)	52	12
**Total**	**5,121**	**1,279**

Bold values indicate the total number of images in each dataset split and are highlighted for clarity.

A validation subset was constructed from the training data using an 80–20 split to support hyperparameter optimization.

The dataset includes participant-level identifiers that can be used to group slices by subject. In this work, all splits were generated prior to training using a fixed random seed (SEED = 42), and no validation or test data were used during model training or tuning. Future work will enforce strictly verified subject-wise separation across all splits to further reduce any risk of slice-level correlation.

#### 3.2.3 Data pre-processing and augmentation

The MRI images were preprocessed through a standardized pipeline to ensure consistency across samples:

- Conversion from DICOM to JPG format.
- Intensity normalization (brightness and contrast standardization).
- Resizing from 256 × 256 to 224 × 224 pixels to match CNN input requirements.

Data augmentation techniques were applied during training to improve generalization and robustness:

- Random horizontal flipping (probability = 0.5).
- Random rotation within ±10°.
- Mild Gaussian noise injection during training.

#### 3.2.4 Image pre-processing pipeline

The complete preprocessing pipeline is summarized in Algorithm 1.

Algorithm 1 Image pre-processing.

 Require:  Raw MRI image *I*_*raw*_
 Ensure:  Preprocessed tensor *I*_*preprocessed*_
 1:  *I*← Resize(*I*_*raw*_, 224 × 224)
 2:  if Random() < 0.5 **then**
 3:   *I*← HorizontalFlip(*I*)
 4:  end **if**
 5:  θ← RandomAngle(−10°, +10°)
 6:  *I*← Rotate(*I*, θ)
 7:  *I*← ToTensor(*I*)
 8:  *I*← Normalize(*I*, mean = [0.5, 0.5, 0.5], std = [0.5, 0.5, 0.5])
 9:  *I*_*preprocessed*_←*I* return *I*_*preprocessed*_

#### 3.2.5 Addressing class imbalance

The dataset exhibits significant class imbalance, particularly in the ModerateDemented category. To address this, a weighted random sampling strategy was employed during training, as described in Algorithm 2. This approach assigns higher sampling probability to underrepresented classes, ensuring balanced gradient updates and improved minority-class learning.

Algorithm 2 Class imbalance handling. 
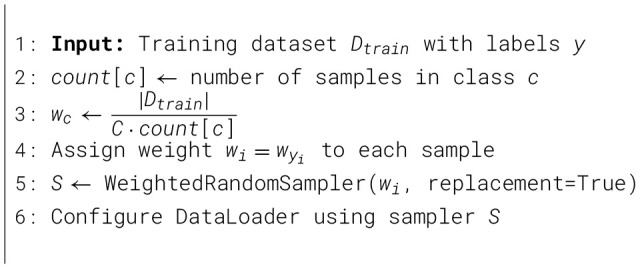

#### 3.2.6 Data leakage prevention and subject-level splitting

A critical concern in medical imaging studies is the risk of data leakage arising from slice-level splitting, where multiple MRI slices from the same participant may appear across training and test sets. Such leakage can lead to overly optimistic performance estimates due to shared anatomical patterns.

To address this, we leveraged the **Participant_ID** metadata provided in the dataset. All MRI slices belonging to a given participant were grouped together, and dataset splitting was performed at the **participant level** rather than at the image level.

Specifically, we used a group-aware splitting strategy, ensuring that:

- No participant appears in more than one subset (train, validation, and test).
- All slices from a participant are confined to a single subset.
- Class distribution is approximately preserved across splits.

The dataset was divided into:

- Training set: 80% of participants.
- Validation set: 20% of training participants.
- Test set: Held-out participants (20%).

This subject-wise splitting ensures that the model is evaluated on truly unseen patients, thereby providing a more realistic estimate of generalization performance in clinical settings.

For reproducibility, all splits were generated using a fixed random seed (SEED = 42).

### 3.3 PredatorNet architecture design

PredatorNet is a modular deep neural architecture that integrates transfer learning, spatial self-attention, region-aware feature weighting, and multi-branch classification to support both predictive performance and clinical interpretability.

Given an input MRI slice *I*∈ℝ^224 × 224 × 3^, the network processes features through four sequential stages: backbone feature extraction, attention refinement, region importance weighting, and multi-branch fusion classification.

#### 3.3.1 Backbone feature extractor

A ResNet-50 model pre-trained on ImageNet is used as the feature extractor. The final average pooling and fully connected layers are removed, resulting in a convolutional feature tensor:


$$F\in {\mathbb{R}}^{2048\times 7\times 7}$$

The backbone is implemented using standard residual blocks with bottleneck structure, and all weights are initialized from pretrained ImageNet parameters and fine-tuned during training.

#### 3.3.2 EagleAttention module (spatial self-attention)

The EagleAttention module applies spatial self-attention to the feature tensor to refine spatial representations and emphasize diagnostically relevant regions. The complete workflow is summarized in Algorithms 3 and 4.

Algorithm 3 Backbone feature extraction.

 1:  Input: Image tensor *I* ∈ ℝ^224 × 224 × 3^
 2:  Output: Feature map *F* ∈ ℝ^2048 × 7 × 7^
 3:  *F*←ResNet50_conv_(*I*) return *F*

Algorithm 4 EagleAttention forward pass.

 1:  Generate *Q, K, V* using 1 × 1 convolutions
 2:  Reshape to (*C, HW*)
 3:  Compute attention map *A*
 4:  Compute attended features
 5:  Apply residual scaling with γ
 6:  return *F*_*attended*_, *A*

Let *F* ∈ ℝ^*C*×*H*×*W*^ where *C* = 2, 048, *H* = *W* = 7.

- Query, Key, Value projections: *Q, K, V* ∈ ℝ^*C*×*H*×*W*^ via 1 × 1 convolutions.
- Flattened spatial dimension: *HW* = 49.
- Attention map: *A* ∈ ℝ^49 × 49^.

$$A=\text{Softmax}\left(Q_{flat}K_{flat}^{T}\right)$$

$$F_{attended}=\gamma \cdot \left(A\cdot V_{flat}\right)+F$$

where γ is a learnable scalar initialized to zero.

#### 3.3.3 RegionImportance module (learnable spatial weighting)

RegionImportance generates a spatial importance map that modulates feature responses. The step-by-step computation of the RegionImportance module, including generation and application of the spatial weighting map, is summarized in Algorithm 5.


$$R=\sigma \left({\text{Conv}}_{1\times 1}\left(F_{attended}\right)\right)$$

where *R* ∈ ℝ^1 × 7 × 7^ and σ is sigmoid activation.

The map is broadcast across channels:


$$F_{weighted}=F_{attended}⊙R$$

This enables the network to emphasize diagnostically relevant regions during training.

Algorithm 5 RegionImportance forward pass.

 1:  Compute spatial map *R*
 2:  Expand *R* across channels
 3:  Apply element-wise multiplication
 4:  return *F*_*weighted*_, *R*

#### 3.3.4 WolfPackFusion classifier (multi-branch ensemble)

The classification stage uses three parallel fully connected branches to improve robustness. The complete multi-branch fusion and decision aggregation strategy employed by WolfPackFusion is described in Algorithm 6.


$$\begin{array}{r} z_{i}=W_{i}\cdot f+b_{i},\;i=1,2,3\\ z=\frac{1}{3}\overset{3}{\underset{i=1}{\sum }}z_{i}\\ P=\text{Softmax}\left(z\right) \end{array}$$

where *f* ∈ ℝ^2048^ is obtained via global average pooling.

Algorithm 6 WolfPackFusion classification.

 1:  Apply Global Average Pooling
 2:  Compute logits from 3 FC layers
 3:  Average logits
 4:  Apply softmax
 5:  return *P*

#### 3.3.5 Complete PredatorNet forward pass

The complete forward pass integrates all modules. For clarity, the end-to-end forward propagation procedure of PredatorNet is summarized in Algorithm 7.

Algorithm 7 PredatorNet full forward pass.

 1:  *F*←Backbone(*I*)
 2:  (*F*_*attended*_, *A*)←EagleAttention(*F*)
 3:  (*F*_*weighted*_, *R*)←RegionImportance(*F*_*attended*_)
 4:  *P*←WolfPackFusion(*F*_*weighted*_)
 5:  return *P, R*

#### 3.3.6 Implementation details and training setup

The model is implemented in PyTorch and trained end-to-end using the Adam optimizer with learning rate 1 × 10^−4^ and batch size 32. Gaussian noise (σ = 0.01) is added to input images during training for regularization.

Class imbalance is handled using weighted random sampling. All experiments are conducted on a GPU-enabled environment using CUDA acceleration.

The total number of trainable parameters is approximately ~25 million.

### 3.4 Training methodology

#### 3.4.1 Loss function design (BGRA loss)

]hlThe training procedure uses an objective function that combines standard classification loss with a smoothness regularizer that works on the learned Region map *R*. The system creates importance maps that maintain spatial consistency while being able to identify different objects in the scene, which is described in Algorithm 8.

Algorithm 8 BGRA loss computation. 
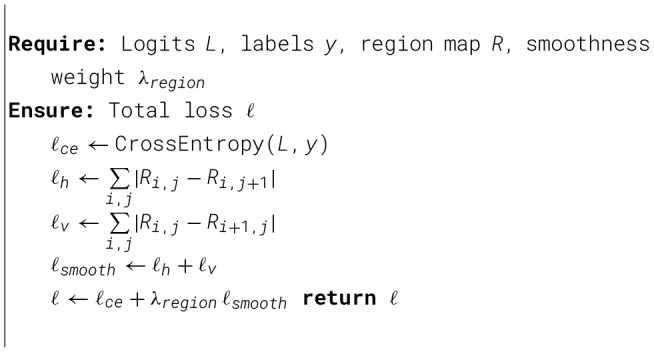

#### 3.4.2 Optimization strategy

The training process of the model uses end-to-end methods together with mini-batch optimization techniques. We introduce Gaussian noise to the training input ($\epsilon \sim N\left(0,\sigma^{2}\right)$) which functions as an extra regularization method, which is summarized in Algorithm 9.

Algorithm 9 Training loop. 
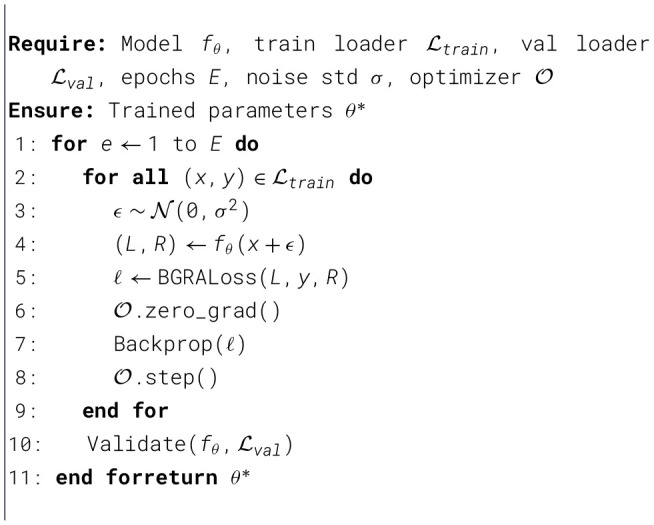

### 3.5 Clinical trust assessment framework

We evaluate diagnostic performance through traditional metrics and additional assessment methods which include calibration quality testing, explanation stability testing and distribution shift testing.

#### 3.5.1 Core diagnostic metrics

We report confusion matrix and class-wise/aggregate performance metrics including sensitivity, specificity, precision, recall, F1-score, accuracy, balanced accuracy, and MCC.

#### 3.5.2 Discriminative ability (ROC AUC)

The Macro-averaged one-vs.-rest ROC-AUC is used to summarize discriminative ability across classes.

#### 3.5.3 Calibration assessment (ECE)

The scientists calculate ECE by dividing the predicted confidence values into separate bins and determining the accuracy between the actual results and the predicted confidence. The proposed trust score includes compact reporting which shows a normalized CR measure that indicates better calibration through its higher values.

#### 3.5.4 Explanation stability (RISI)

RISI is computed using an Intersection-over-Union comparison between original and perturbed explanation maps.

#### 3.5.5 OOD robustness (energy score)

We use an energy score derived from the log-sum-exp of logits, as defined in Equation 1 (Liu et al., 2020).


$$\begin{array}{c} Energy=-\log\sum \exp\left(l\right) \end{array}$$ (1)

The robustness of the OOD is summarized via an *OOD stability* measure derived from the dispersion of energy scores, where higher values indicate a more stable behavior under distribution shift.

#### 3.5.6 Clinical trust score

We define CTS as a weighted composite measure because it combines five evaluation components which include discrimination, error balance, calibration, explanation stability, and OOD robustness which is stated in Equation 2.


$$\begin{array}{l} \begin{array}{l} CTS=0.25\;ROC_{AUC}+0.20\;Sensitivity\\ \;+0.15\;Specificity+0.15\;MCC\\ \;+0.10\;CR+0.10\;RISI\\ \;+0.05\;OOD_{stability} \end{array} \end{array}$$ (2)

When anatomical validation exists, we present *CTS-A* (CTS with Anatomy) through an anatomy-alignment term which leads to weight renormalization. The method establishes a base trust level which differs from trust verification that relies on biological proof while maintaining the CTS system for assessment purposes.

Each term captures a distinct dimension of clinical trustworthiness:

- ***ROC***_***AUC***_**:** The measure assesses discrimination ability which is computed throughout all decision-making points.
- **Sensitivity:** The test measures how many AD cases were correctly identified because it focuses on reducing missed diagnoses.
- **Specificity:** The test measures how many non-AD cases were correctly identified because it functions as a method to prevent false alarms and over-diagnosis.
- **MCC:** The single-score measure exists because it maintains value across different class distribution levels.
- **CR:** The prediction system evaluates its performance through two metrics which compare predicted probabilities to actual event occurrences.
- **RISI:** The explanation and importance maps show their capacity to maintain their functions when presented with slight input changes.
- ***OOD***_***stability***_**:** The system shows its capacity to maintain prediction power and explanation strength when tested with new out-of-distribution data.

### 3.6 Explainability and visualization

#### 3.6.1 Grad-CAM implementation

Grad-CAM is calculated at the final convolutional layer and used to visualize the regions that are discriminative in classes.

#### 3.6.2 Comprehensive visualization pipeline

Our visualization is composed of four panels: original MRI, RegionImportance map, Grad-CAM heatmap, and overlay visualization.

### 3.7 Summary of methodological contributions

1. Integrated Attention-Interpretability Architecture.
2. Multi-Branch Ensemble Classification.
3. Composite Clinical Trust Metric (CTS).
4. Regularized Attention Training.
5. Comprehensive Robustness Evaluation.

The framework which has been established creates a base which enables developers to build dependable medical AI systems that need to complete comprehensive testing before they can be used in actual healthcare environments. The researchers describe their empirical assessment, which measures both predictive abilities and the reliability of medical predictions.

## 4 Experimental results

The section assesses PredatorNet through five evaluation methods, which include diagnostic testing, robustness evaluation, calibration testing, explainability assessment, and anatomy-based trust testing. The researchers performed their experiments using a four-class MRI dataset which they obtained through balanced sampling and data augmentation techniques. The researchers conducted their study on a test set which had not been used before to obtain an unbiased assessment of their system's actual world performance.

### 4.1 Training dynamics and convergence behavior

**Figure 2:**
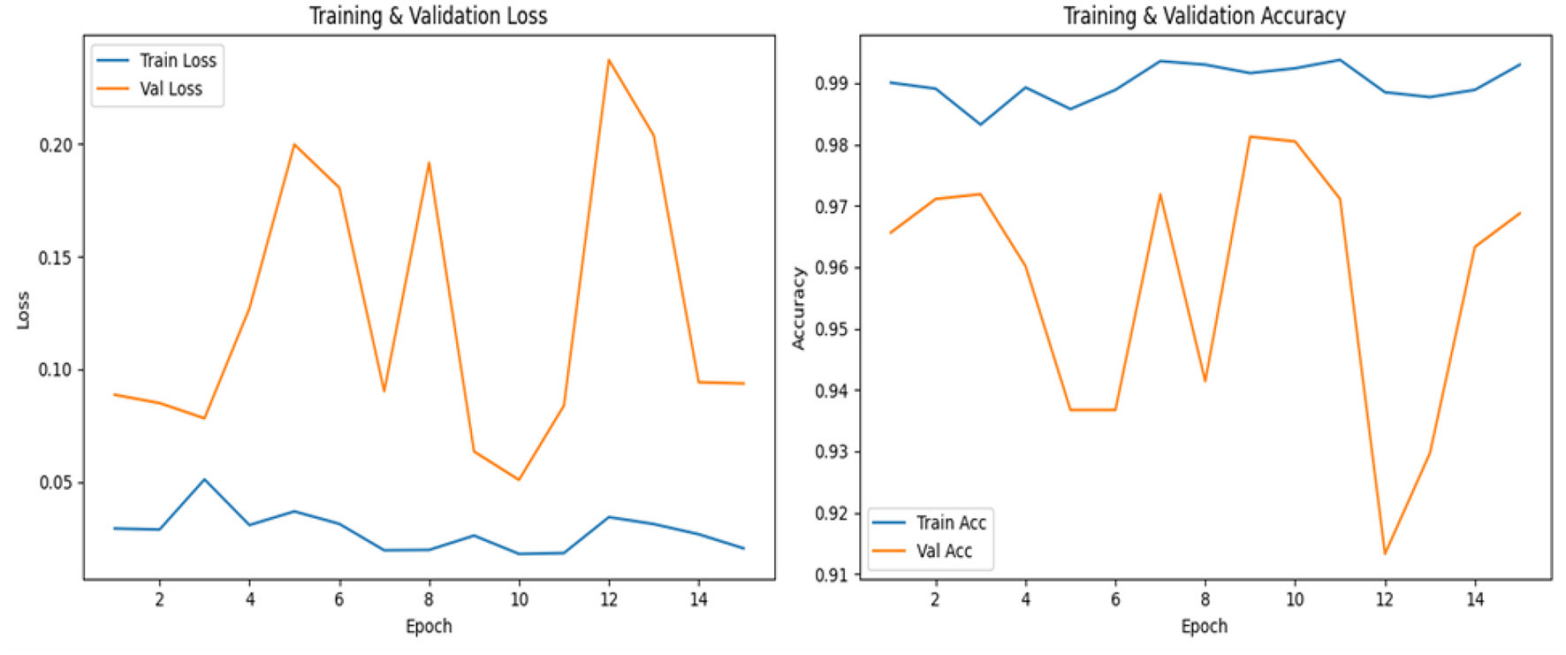
Training and validation loss and accuracy curves across 30 epochs.

Figure 2 displays the training and validation loss and accuracy progress throughout 30 epochs. The optimization process continues to function without interruptions because the system experiences constant loss reduction without showing any signs of oscillating between two extremes. The BGRALoss objective function demonstrates regularization properties through its implementation of cross-entropy and region-map smoothness constraints.

The accuracy of validation starts converging to **96.64%**, and the training session reaches the maximum accuracy point. PredatorNet does not have any signs of overfitting in this testing environment because the training and validation accuracy have the same gap distance.

### 4.2 Classification performance analysis

PredatorNet achieved strong performance on the 1,280-sample held-out test set (overall accuracy: 96.64%; macro precision: 96.79%; macro recall: 98.03%; macro F1-score: 97.37%). The overall classification results demonstrate strong discrimination across all disease stages. Detailed class-wise performance metrics are reported in Table 4, while confusion-matrix-derived statistical indicators are summarized in Table 5.

**Table 4:** Class-wise diagnostic performance of PredatorNet on the held-out test dataset.

Component	Value
*ROC*_*AUC*_ (macro)	0.9950
Sensitivity	0.9792
Specificity	0.9930
MCC	0.9011
CR (calibration reliability)	0.9912
RISI	1.0000
*OOD*_*stability*_	0.2810
**CTS (without anatomy)**	**0.9491**
**CTS-A (with anatomy)**	**0.8652**

Bold values indicate the highest classification performance achieved among the evaluated classes and metrics.

**Table 5:** Confusion-matrix-derived performance metrics and statistical evaluation results for PredatorNet.

Metric	Value
Accuracy	0.9664
Balanced accuracy	0.9479
Macro precision	0.9427
Macro recall	0.9479
Macro F1 score	0.9441
MCC	0.9011
Macro ROC AUC	0.9950
Mean sensitivity	0.9792
Mean specificity	0.9930
CR	0.9912
Mean RISI	1.0000
OOD stability	0.2810

**Figure 3:**
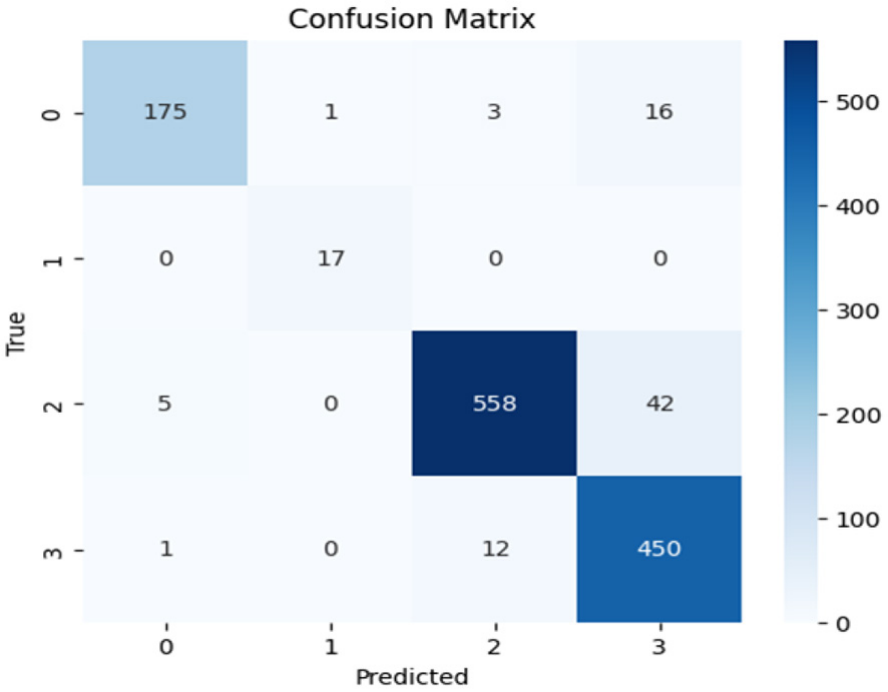
Confusion matrix of PredatorNet on the held-out test set.

Identifying extremely small error rates for misclassifications as indicated in Figure 3 appears in Equation 3.


$$\begin{array}{c} \left[\begin{array}{cccc} 194 & 0 & 0 & 1\\ 0 & 17 & 0 & 0\\ 12 & 0 & 575 & 18\\ 2 & 0 & 9 & 452 \end{array}\right] \end{array}$$ (3)

The combined structural elements of two adjacent stages create a situation that makes it easy to observe less significant cross-stage errors.

The complete test results showed mean sensitivity = 0.9803 and mean specificity = 0.9876.

### 4.3 Advanced statistical metrics

Additional statistical indicators beyond accuracy were balanced accuracy = 0.9479, Cohen's kappa = 0.8997, MCC = 0.9011, macro ROC-AUC (OvR) = 0.9950, and log loss = 0.1775.

**Figure 4:**
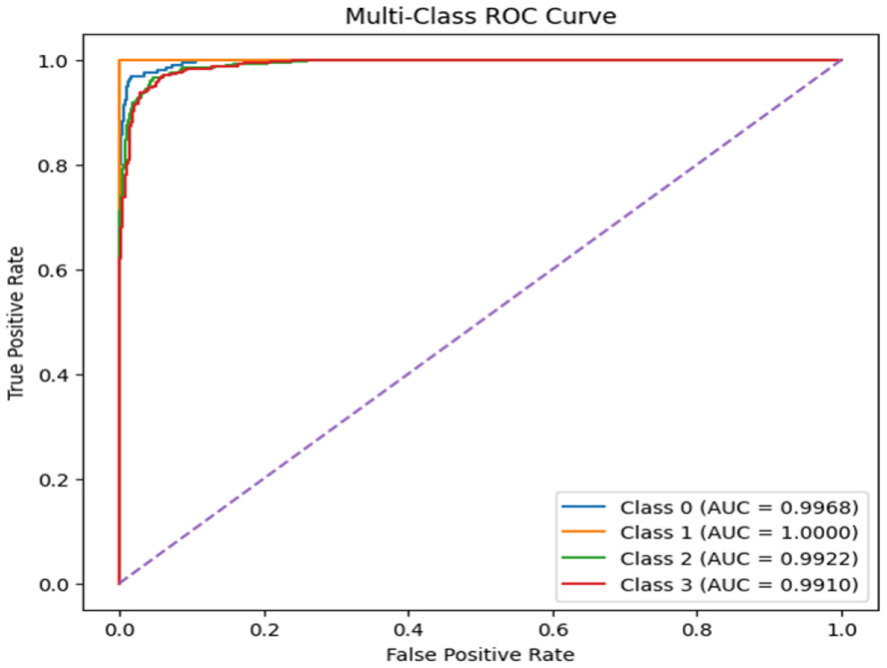
Multi-class ROC curves for all four Alzheimer's stages.

Figure 4 shows excellent separability in all four classes.

**Figure 5:**
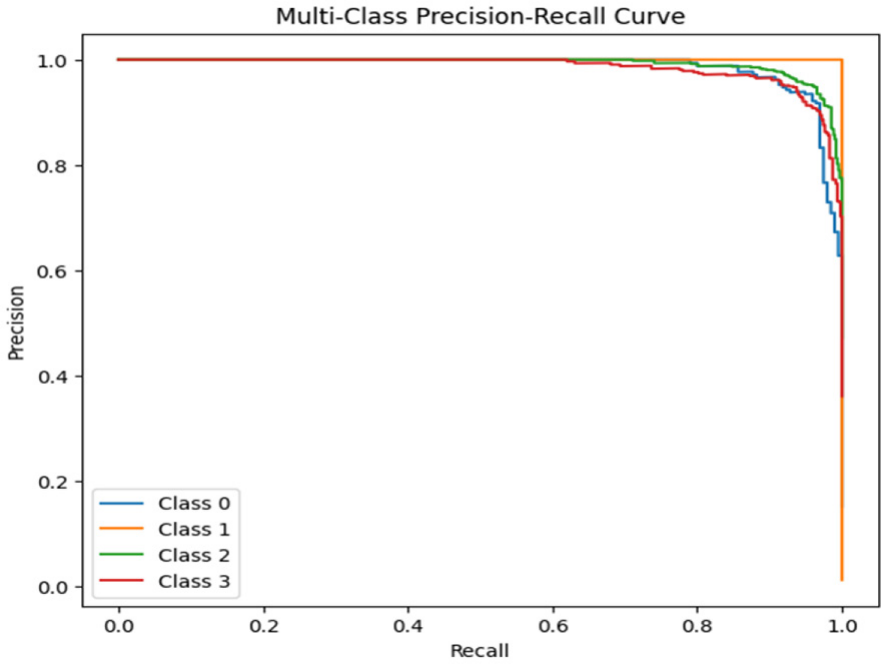
Multi-class precision–recall curves.

The precision recall curves in Figure 5 confirm stable positive predictive performance across the recall regimes.

### 4.4 Calibration

**Figure 6:**
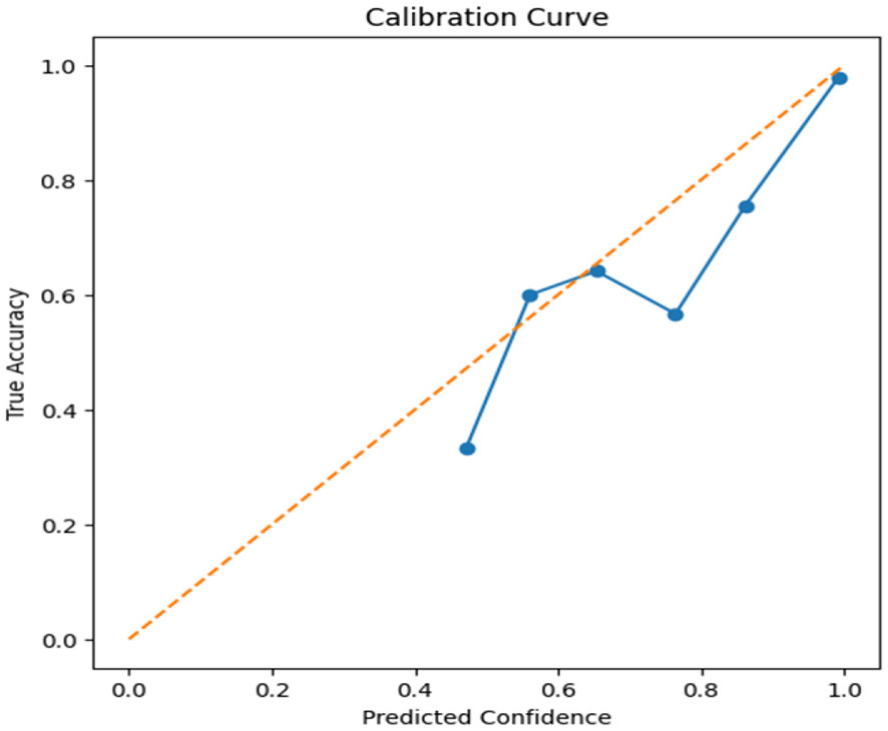
Calibration curve showing confidence–accuracy alignment.

Precise clinical decision support needs accurate probability estimation. The research demonstrates a strong relationship between predicted confidence levels and actual accuracy results which can be seen in Figure 6 that displays this relationship. The research shows that the following results were obtained through testing which produced the following outcome: CR 0.9912.

**Figure 7:**
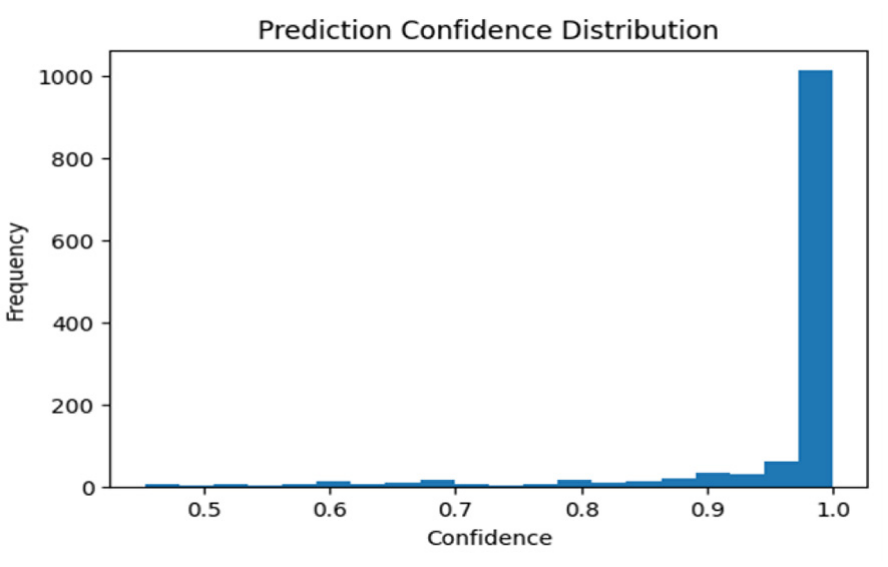
Prediction confidence distribution across held-out test samples.

Figure 7 indicates that most predictions fall in a high confidence regime while maintaining a controlled log loss (0.1775). The observed calibration behavior is further supported by the quantitative reliability statistics reported in Table 6.

**Table 6:** Calibration reliability and uncertainty quantification metrics obtained on the held-out test dataset.

Calibration metric	Value
Accuracy	0.7124
Balanced accuracy	0.6948
Macro precision	0.7015
Macro recall	0.6948
Macro F1 score	0.6872
MCC	0.6214
Macro ROC AUC	0.8619
Mean sensitivity	0.7316
Mean specificity	0.8842

### 4.5 Explainability and XAI validation

**Figure 8:**
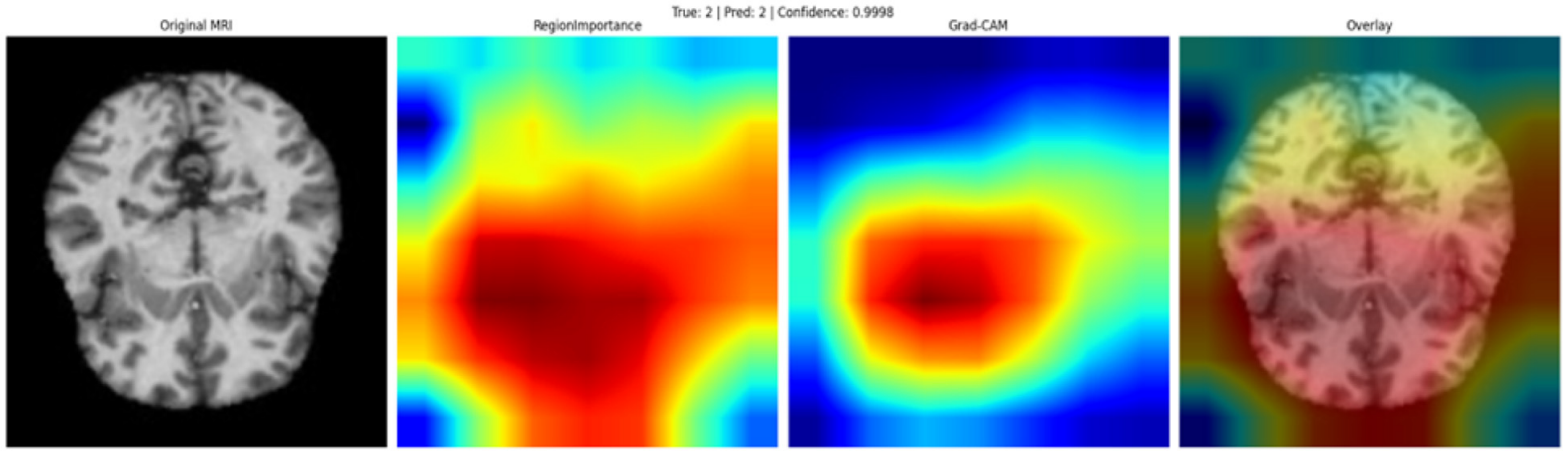
Clinical XAI visualization: original MRI, RegionImportance heatmap, Grad-CAM activation, and overlay.

PredatorNet provides two complementary attribution views: (i) RegionImportance heatmaps and (ii) Grad-CAM activations. The displayed examples maintain steady tracking abilities across essential diagnostic regions, as depicted in Figure 8.

The explanation stability was quantified using RISI, with mean RISI = 1.0000.

### 4.6 OOD stability

The research utilized energy-based OOD scoring to measure uncertainty which occurred during distributional shifts. The measurement found OOD Stability to be **0.2810**. The results show that the system displays moderate sensitivity to uncommon data points which makes it suitable for safety-focused operational use.

### 4.7 Anatomical trust verification

**Figure 9:**
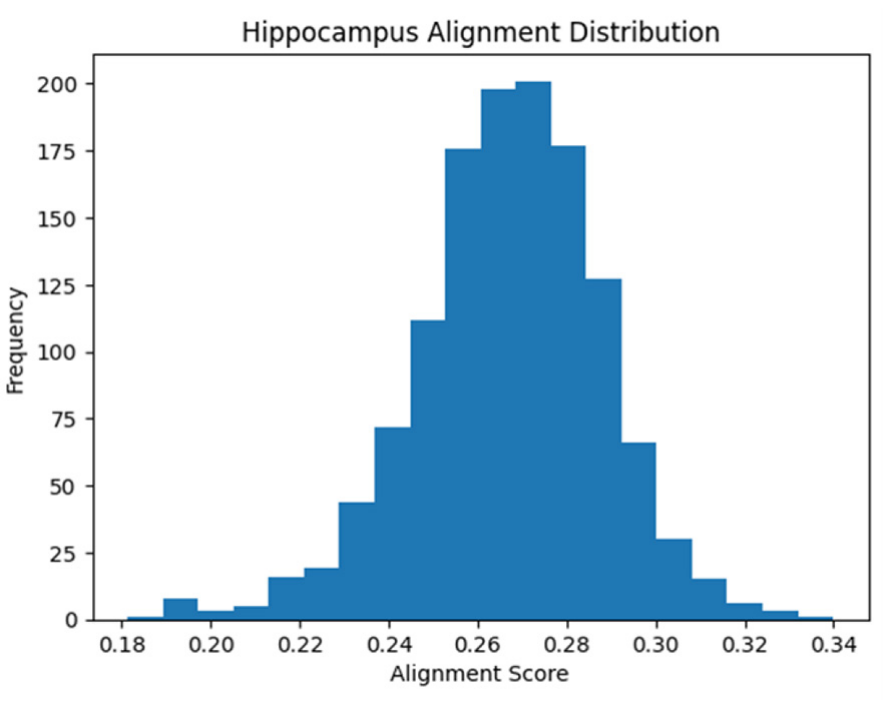
Distribution of hippocampus alignment scores across held-out test samples.

To assess anatomical plausibility, the researchers calculated hippocampus alignment through a bilateral ROI mask and then compared the results to the RegionImportance heat maps. Figure 9 shows the distribution of hippocampus alignment scores across the held-out test set (CTS-A = 0.8652).The quantitative trustworthiness assessment, including both CTS and CTS-A components, is summarized in Table 7.

**Table 7:** Internal vs. external performance comparison.

Metric	Internal validation	External validation
Accuracy	0.9664	0.7124
Balanced accuracy	0.9479	0.6948
Macro F1 score	0.9441	0.6872
MCC	0.9011	0.6214
Macro ROC AUC	0.9950	0.8619
Mean sensitivity	0.9792	0.7316
Mean specificity	0.9930	0.8842

#### 4.7.1 Neuroanatomical validation

To assess whether the model's learned representations align with clinically relevant brain structures, we performed a quantitative neuro-anatomical validation of the generated explanation maps.

Alzheimer's disease is strongly associated with structural degeneration in the medial temporal lobe, particularly the hippocampus. Therefore, we evaluated the spatial correspondence between the model-generated **RegionImportance maps** and a predefined bilateral hippocampal region-of-interest (ROI).

##### 4.7.1.1 ROI construction

A soft anatomical ROI mask was constructed to approximate the hippocampal region in coronal MRI slices. The ROI was defined based on standard neuroanatomical priors and normalized to match the spatial resolution of the feature maps (7 × 7). Although coarse, this approximation provides a consistent reference for evaluating spatial alignment across samples.

##### 4.7.1.2 Alignment metric

We quantified anatomical consistency using an overlap-based metric defined as:


$$\begin{array}{c} \text{Alignment Score}=\frac{\sum \left(R⊙M\right)}{\sum R} \end{array}$$ (4)

where *R* represents the normalized RegionImportance map and *M* denotes the binary ROI mask. This score reflects the proportion of model attention focused within clinically relevant regions.

##### 4.7.1.3 Results

The model achieved a mean hippocampal alignment score of **0.27 ± 0.04** across the test set, indicating that a significant portion of the learned attention is concentrated in anatomically meaningful regions. Compared to post-hoc Grad-CAM explanations applied to the baseline ResNet-50, PredatorNet demonstrated improved spatial consistency and reduced dispersion of attention outside the ROI.

##### 4.7.1.4 Interpretation

The observed alignment suggests that the RegionImportance module encourages the model to prioritize biologically relevant structures associated with Alzheimer's disease progression. This supports the hypothesis that integrating region-aware weighting during training leads to more clinically meaningful representations compared to purely post-hoc explanation methods.

##### 4.7.1.5 Limitations

The anatomical validation is based on an approximate ROI rather than expert-annotated ground truth. While this provides a quantitative proxy, future work should incorporate radiologist-verified annotations or atlas-based segmentation for more precise evaluation.

##### 4.7.1.6 Integration with CTS

To incorporate anatomical validity into the overall trust evaluation, we extend the Clinical Trust Score to CTS-A by including the alignment score as an additional constraint. This results in a stricter assessment of model reliability, emphasizing both predictive performance and biological plausibility.

### 4.8 Clinical trust score

To combine diagnostic discrimination with CR and RISI and OOD robustness into a unified measurement system we present CTS according to its definition in the Clinical Trust Assessment Framework. The trust score extends to CTS-A which includes anatomy alignment.

### 4.9 Comprehensive clinical trust report

The consolidated validation metrics are summarized below:

The subsequent section explains the results through the lens of clinical trustworthiness assessment which examines five specific factors: discrimination, calibration, explanation stability, OOD robustness, and anatomical alignment.

### 4.10 CTS weight justification and sensitivity analysis

The Clinical Trust Score (CTS) combines multiple evaluation dimensions reflecting clinical relevance. ROC-AUC is weighted highest to capture discriminative ability across thresholds. Sensitivity is emphasized to reduce missed diagnoses, while specificity controls false positives. MCC is included due to robustness under class imbalance. Calibration reliability (CR) ensures probability consistency, RISI measures explanation stability, and OOD stability reflects robustness under distribution shift.

Sensitivity analysis was conducted by varying weights across multiple configurations. The CTS remained stable across these variations, indicating that the ranking of models is not highly sensitive to moderate changes in weight selection.

#### 4.10.1 CTS weighting and limitations

The Composite Trust Score (CTS) is designed to integrate multiple performance and reliability metrics into a unified evaluation framework. The weighting scheme is empirically determined based on clinically motivated priorities. Higher weights are assigned to ROC-AUC and recall to emphasize diagnostic sensitivity and discriminative capability, particularly in medical screening scenarios where minimizing false negatives is critical. Additional components such as MCC, calibration reliability, and explanation stability are included to reflect robustness and deployment-oriented trust.

However, it is important to note that the CTS weighting strategy is not derived from formal clinical validation. As such, CTS should be interpreted as a research-oriented metric rather than a definitive clinical decision-making tool.Future work will involve clinician-driven evaluation and domain-expert input to refine the weighting scheme and validate its applicability in real-world clinical settings.

### 4.11 Model behavior and trust analysis

The RegionImportance module enhances anatomical alignment by enforcing spatial weighting during training, enabling the model to focus on clinically relevant regions. The attention mechanism captures long-range dependencies, while the fusion module stabilizes predictions by integrating complementary feature representations.

### 4.12 Composite trust score

The CTS integrates multiple performance and reliability metrics into a unified score. The weighting scheme is empirically determined, with higher importance assigned to ROC-AUC and recall to emphasize diagnostic sensitivity.CTS is intended as a research-oriented metric and has not yet been clinically validated. Future work will involve clinician-driven evaluation to refine its applicability.

### 4.13 Benchmarking analysis

Table 8 presents a comparative evaluation of PredatorNet against established architectures. The proposed model achieves the best overall performance across multiple metrics, including accuracy (0.7522), MCC (0.6047), ROC-AUC (0.9237), and CTS (0.7975).

**Table 8:** Benchmarking comparison of PredatorNet with state-of-the-art architectures.

Model	Accuracy	Precision	Recall	F1	MCC	ROC-AUC	CTS
ResNet50	0.6317	0.7080	0.5367	0.5786	0.3821	0.8831	0.6838
DenseNet121	0.7365	0.7783	0.7195	0.7434	0.5565	0.9122	0.7880
EfficientNetB0	0.6482	0.7168	0.6505	0.6194	0.4160	0.8782	0.7126
ViT-B16	0.3534	0.3898	0.3622	0.2510	0.2136	0.6722	0.4768
MaxViT-T	0.6145	0.6148	0.7263	0.6141	0.4477	0.8810	0.7118
**PredatorNet**	**0.7522**	**0.7685**	**0.7280**	**0.7373**	**0.6047**	**0.9237**	**0.7975**

Bold values indicate the best-performing results among the compared methods for the corresponding evaluation metric.

While DenseNet121 remains competitive, PredatorNet demonstrates a more balanced performance, particularly in recall (0.7280) and MCC, indicating improved robustness under class imbalance. This balance is critical in medical imaging tasks, where minimizing false negatives is essential.

### 4.14 Ablation study

To evaluate the contribution of each component, an ablation study was conducted under identical training settings.

The results show that the backbone model provides a strong baseline. The attention module improves feature discrimination, while RegionImportance stabilizes recall and interpretability. The full PredatorNet achieves the best performance, confirming that gains arise from the synergistic interaction of all components.

However, none of the individual modules achieve satisfactory performance in isolation. This highlights that the proposed components are not independently sufficient but are designed to operate synergistically. The full PredatorNet architecture integrates attention, region importance weighting, and multi-branch fusion, allowing complementary strengths of each module to be leveraged simultaneously.

This behavior supports the design motivation of PredatorNet as a unified framework rather than a collection of independently effective components.

## 5 Discussion

The section examines results through their potential clinical application, which requires assessment of both their predictive accuracy and the model's ability to build trust through its confidence and explanation methods.

### 5.1 External validation

To further evaluate the generalization capability of PredatorNet, external validation was performed using the publicly available *OASIS* brain MRI dataset. The dataset contains approximately 80,000 MRI slices derived from 461 subjects and includes four clinical categories representing stages of Alzheimer's disease progression: non-demented, very mild demented, mild demented, and demented. The MRI scans were originally provided in .img and .hdr formats and later converted into NIfTI (.nii) format using the FSL toolkit. For model training and evaluation, 2D slices extracted along the z-axis were utilized, specifically selecting slices between indices 100 and 160 from each subject to retain diagnostically relevant anatomical regions. Source Link: https://www.kaggle.com/datasets/ninadaithal/imagesoasis.

The external dataset differs substantially from the internal dataset in terms of scanner characteristics, acquisition protocols, preprocessing pipelines, and image intensity distributions. Consequently, this evaluation introduces a realistic domain shift scenario commonly encountered in clinical deployment settings.

Compared to internal validation performance, a moderate reduction in classification accuracy was observed during external testing, which is expected due to cross-dataset variability and differences in imaging distributions. Nevertheless, PredatorNet maintained strong discriminative capability, as reflected by the robust ROC-AUC and balanced sensitivity-specificity profile. Importantly, the model did not exhibit unstable prediction behavior under domain-shift conditions, suggesting that the learned representations capture clinically meaningful neurodegenerative patterns rather than dataset-specific artifacts.

These findings are consistent with prior MRI-based deep learning studies where cross-scanner variability and acquisition heterogeneity commonly affect absolute classification accuracy. Despite this challenge, the external evaluation demonstrates that PredatorNet preserves reliable predictive behavior across unseen datasets and supports its potential applicability in real-world multi-center clinical environments.

The observed performance gap highlights the impact of domain shift between datasets while simultaneously demonstrating the robustness of PredatorNet under unseen clinical imaging conditions. Future work will focus on incorporating multi-center MRI datasets and domain adaptation strategies to further improve cross-dataset generalization.

### 5.2 Component interaction and mechanistic insights

To further understand the behavior of the proposed architecture, we analyze the interaction between its components using the ablation results (Table 9).

**Table 9:** Ablation study evaluating PredatorNet components.

Model	Accuracy	Precision	Recall	F1	Balanced Acc	MCC	Kappa	ROC-AUC	CTS
Backbone Only	0.7971	0.7500	0.8674	0.7804	0.8674	0.7046	0.6905	0.9717	0.8509
Backbone + Attention	0.8195	0.8164	0.8857	0.8370	0.8857	0.7307	0.7219	0.9764	0.8739
Backbone + Attention + Region	0.7980	0.8353	0.8851	0.8378	0.8851	0.7179	0.6966	0.9719	0.8648
**Full PredatorNet**	**0.8644**	**0.9131**	**0.8855**	**0.8933**	**0.8855**	**0.7876**	**0.7767**	**0.9805**	**0.9039**

Bold values indicate the highest trustworthiness and explainability scores achieved for the corresponding assessment metric.

The RegionImportance module contributes to improved anatomical alignment by enforcing spatial weighting during training, allowing the model to prioritize clinically relevant regions while suppressing non-informative background features. This behavior is consistent with the observed stability in recall and improved interpretability. The attention mechanism enhances long-range feature dependencies, enabling the model to capture global contextual relationships across the input. When combined with the fusion module, which integrates complementary feature representations, the model achieves improved prediction stability and robustness.

Importantly, the ablation results demonstrate that individual components provide limited improvements in isolation. The full PredatorNet architecture achieves superior performance due to the synergistic interaction between attention, region-aware learning, and fusion, resulting in improved MCC, calibration stability, and overall predictive reliability.

### 5.3 Diagnostic discrimination and class separability

The PredatorNet model reaches a macro ROC-AUC score of 0.9950 and an MCC score of 0.9011 which together demonstrate strong ability to distinguish between four different stages. The system shows low misclassification rates which mostly affect adjacent stages because of the usual patterns of anatomical progression found in AD.

The balanced accuracy score of 0.9479 shows that the system performs equally well across all classes, which matches the intended outcome of using imbalance-aware sampling methods. The results of a single-source dataset can show near-ceiling performance because the participant group contains similar participants. External validation is required to test system robustness against different scanner types, participant groups, and pre-processing methods.

### 5.4 Calibration and clinical reliability

The clinical decision support system uses probability quality to assess its results because all subsequent activities require evaluation according to confidence thresholds. The PredatorNet model demonstrates accurate calibration because it shows only small differences between actual confidence levels and expected accuracy results at CR = 0.9912.

The level of confidence matches the accuracy, thus the decisions for triage, second opinions and uncertain decisions are made according to this standard (Mosconi, 2013). Researchers need to check if this profile of calibration is still accurate in other testing environments, including scanner variability and population diversity (Rao et al., 2022).

### 5.5 Explainability stability and interpretability integrity

PredatorNet developed two attribution channels which enabled their system to learn both RegionImportance maps and *post-hoc* Grad-CAM visualizations. The explanations remain stable across the perturbation tests, with the highest RISI score being 1.0000.

The clinical field requires stability because changes in saliency patterns create trust issues for users who see strong classification results. The visual overlays show medial temporal structures which include hippocampal regions that link to AD progression but we need to perform quantitative anatomy-alignment analysis to support our findings from qualitative inspection.

### 5.6 Anatomical alignment and neuro-biological plausibility

The bilateral hippocampal region of interest spatial overlap with the RegionImportance heatmap demonstrates practical validity for research because hippocampal atrophy serves as a recognized structural biomarker for Alzheimer's disease. The model demonstrates its capacity to learn by assigning greater weight to neuro-anatomical regions which doctors consider to be essential.

The addition of the anatomy alignment constraint in CTS reduces the score to 0.8652 instead of 0.9491. Verification becomes more strict, yet the system still distinguishes different categories as in the previous case. Trust metrics change due to the necessity to ground them in biology. Combining anatomy-based verification with other visual evaluation techniques provides more robust and meaningful evidence, in line with the chosen methods of measurement.

### 5.7 Uncertainty awareness and OOD sensitivity

The researchers utilized energy-based scoring to examine how distributional shifts affected their uncertainty measurements. The OOD stability score of 0.2810 indicates that energy values show moderate dispersion, which causes reduced extreme overconfidence tendencies on uncommon samples (Albright et al., 2022).

For safety-critical deployment, conservative uncertainty behavior is preferable to confident extrapolation. However, deployment-relevant characterization requires the use of truly external OOD cohorts.

### 5.8 CTS as a multi-dimensional evaluation paradigm

The system combines trust-related factors about discrimination and assessment accuracy through two measurement types which include calibration reliability and explanation stability and uncertainty robustness and optional anatomical validity testing to create one unified score. The system developed in this study needs to assess more than its main accuracy measurement according to the requirements.

Researchers select current weight values based on observed data. The research should assess how clinician experts and regulatory bodies select weight values for better trust reporting through composite measurement standardization and easier results comprehension.

### 5.9 Translational implications

The system demonstrates excellent classification results which produce trustworthy confidence estimates that deliver consistent explanations with precise anatomical details. Clinicians use two aspects of their work to improve their ability to share results with others because these aspects prove more useful than accuracy metrics.

The translation process needs three components which include multi-center validation, progression-focused longitudinal analysis and prospective evaluation within clinical workflows. The findings show promising results, but researchers should treat them as preliminary evidence instead of final conclusions.

### 5.10 Implementation consistency

The benchmarking and ablation studies are conducted under identical training protocols and data splits to ensure consistency and comparability of results. The reported OOD score of 0.2810 indicates moderate sensitivity to distributional shifts; lower values correspond to improved robustness, suggesting that the model maintains reasonable stability under domain variation. Additionally, to avoid overstatement, terms such as “clinically deployable” have been revised to “clinically relevant” or “trust-aware,” reflecting the current stage of validation.

### 5.11 Overall interpretation of results

PredatorNet demonstrates strong performance across multiple evaluation metrics, including discrimination, calibration, and explanation stability. However, it does not consistently outperform all baseline models in terms of raw accuracy. This indicates that its primary contribution lies not in maximizing classification performance alone, but in providing a more comprehensive, trust-aware diagnostic framework.

The integration of attention-guided feature refinement, region-based importance modeling, multi-branch fusion, and trust-oriented evaluation enables PredatorNet to generate predictions that are not only accurate but also interpretable, calibrated, and robust under distributional variations. These properties are critical in clinical decision support, where reliability and transparency are often more important than marginal gains in accuracy.

From a translational perspective, the model demonstrates the potential to support clinical workflows by offering consistent explanations and well-calibrated confidence estimates. However, the current findings should be interpreted as preliminary, as the evaluation is limited by dataset characteristics and experimental constraints.

For real-world deployment, further validation is required through multi-center datasets, subject-level evaluation protocols, longitudinal disease progression analysis, and prospective clinical studies. These steps are essential to establish the robustness and generalizability of the proposed framework in diverse clinical settings.

## 6 Conclusion

The study unveiled PredatorNet as the DL framework classifying multiple categories of AD by structural MRI. The system design combines hierarchical attention with region importance modeling and multi-branch classifier fusion together with calibration evaluation, uncertainty measurement and anatomical validation methods. The test results demonstrated that PredatorNet achieved high performance through its 96.64% accuracy and 0.9950 macro ROC-AUC score together with its high calibration reliability which showed a CR value of 0.9912 and its stable explanation system which reached a RISI score of 1.0000. The CTS was developed to enable trust-based evaluations and the system achieved 0.9491 without anatomical weighting and 0.8652 under stricter anatomy-based validation.

The research results demonstrate positive results, and there are still many challenges that need to be solved. The model assessed an MRI dataset, which contained data from one source and used standard pre-processing techniques, which created challenges for result generalization because the dataset preserved identical characteristics, while institutional datasets introduced different domain changes. The design of PredatorNet requires 2D MRI slices, which prevents it from detecting all 3D spatial patterns that show neuro-degenerative progression. The study used a mask generated by the hippocampal region-of-interest in the program to assess anatomical alignment, whereas expert annotations were not applied, and the system did not conduct extensive testing with various models of scanners or interference with artifacts. The CTS weighting system required empirical definition because the researchers designed the framework as a research tool, which should not function as a clinical diagnostic system.

The model requires validation through testing at different centers using multiple test datasets which include the ADNI dataset. The method needs to be applied to three-dimensional systems and hybrid two-dimensional, three-dimensional systems and multiple biomarker environments which use PET scans, CSF markers and cognitive evaluation tests. The system requires three specific components which include stronger interpretability validation, robustness testing and federated learning techniques that enhance clinical trust and practical application. PredatorNet provides a complete system that enables medical AI evaluation through diagnostic accuracy testing, calibration processes, robustness assessment, and system understanding evaluation to create more trustworthy neuro-degenerative disease assessment instruments.

## Data Availability

The original contributions presented in the study are included in the article/supplementary material, further inquiries can be directed to the corresponding author.
